# Effects of Visual Feedback-Induced Variability on Motor Learning of Handrim Wheelchair Propulsion

**DOI:** 10.1371/journal.pone.0127311

**Published:** 2015-05-20

**Authors:** Marika T. Leving, Riemer J. K. Vegter, Johanneke Hartog, Claudine J. C. Lamoth, Sonja de Groot, Lucas H. V. van der Woude

**Affiliations:** 1 University of Groningen, University Medical Center Groningen, Center for Human Movement Sciences, Groningen, The Netherlands; 2 Amsterdam Rehabilitation Research Center | Reade, Amsterdam, the Netherlands; 3 University of Groningen, University Medical Center Groningen, Center for Rehabilitation, Groningen, The Netherlands; University Zurich, SWITZERLAND

## Abstract

**Background:**

It has been suggested that a higher intra-individual variability benefits the motor learning of wheelchair propulsion. The present study evaluated whether feedback-induced variability on wheelchair propulsion technique variables would also enhance the motor learning process. Learning was operationalized as an improvement in mechanical efficiency and propulsion technique, which are thought to be closely related during the learning process.

**Methods:**

17 Participants received visual feedback-based practice (feedback group) and 15 participants received regular practice (natural learning group). Both groups received equal practice dose of 80 min, over 3 weeks, at 0.24 W/kg at a treadmill speed of 1.11 m/s. To compare both groups the pre- and post-test were performed without feedback. The feedback group received real-time visual feedback on seven propulsion variables with instruction to manipulate the presented variable to achieve the highest possible variability (1^st^ 4-min block) and optimize it in the prescribed direction (2^nd^ 4-min block). To increase motor exploration the participants were unaware of the exact variable they received feedback on. Energy consumption and the propulsion technique variables with their respective coefficient of variation were calculated to evaluate the amount of intra-individual variability.

**Results:**

The feedback group, which practiced with higher intra-individual variability, improved the propulsion technique between pre- and post-test to the same extent as the natural learning group. Mechanical efficiency improved between pre- and post-test in the natural learning group but remained unchanged in the feedback group.

**Conclusion:**

These results suggest that feedback-induced variability inhibited the improvement in mechanical efficiency. Moreover, since both groups improved propulsion technique but only the natural learning group improved mechanical efficiency, it can be concluded that the improvement in mechanical efficiency and propulsion technique do not always appear simultaneously during the motor learning process. Their relationship is most likely modified by other factors such as the amount of the intra-individual variability.

## Introduction

Wheelchair propulsion brings mobility to people with lower-limb disabilities and empowers active community participation [1]. However, wheelchair propulsion is not present in the skill repertoire of most people and often has to be learned in the early stages of the rehabilitation process after disease or injury. Due to the load on the shoulder complex, manual wheelchair propulsion is considered to be a straining form of ambulation and is often associated with overuse injuries of the shoulder [2–5]. The goal of wheelchair propulsion training is to facilitate the motor learning process of this cyclical motor skill, with special consideration for injury prevention.

Motor learning of a cyclical skill, such as wheelchair propulsion, can be seen as an adaptation of the human motor system, which emerges from the interaction between different constraints, and possibly leads to a decrease in energy expenditure [6–9]. Under standardized steady-state submaximal conditions the effect of motor learning in wheelchair propulsion can thus be quantified as a decrease in the energy expenditure and therefore increase in mechanical efficiency, i.e. the ratio of external power output and energy expenditure. On a group level, mechanical efficiency increases during motor learning of wheelchair propulsion [10–13]. However, a recent study reported individual differences in the learning rate of acquiring the new skill of wheelchair propulsion [13]. Concomitant with these differences, a higher within-person (intra-individual) variability of the propulsion technique parameters was shown for the group that increased more in mechanical efficiency, compared to the group that increased less in mechanical efficiency. Therefore it was suggested that this intra-individual variability might have been a property that enhanced the motor learning process [13].

Fundamental motor control studies established that intra-individual variability is not just the product of noise, but that it may facilitate the motor learning process as it improves motor exploration and learner’s adaptability [14, 15]. Recent findings suggest that especially task-relevant variability, and not total variability, is crucial to the performance [16]. In wheelchair propulsion task-relevant variability is expected to be the variability in propulsion technique variables that have previously been associated with mechanical efficiency [12].

With respect to task-relevant variability in wheelchair training, propulsion technique variables such as push frequency, contact angle and braking moment have been shown to be directly related to mechanical efficiency [12]. In addition, it was shown that change within these and other propulsion variables such as fraction effective force, peak force, push distance and smoothness can be targeted by providing visual feedback on these parameters [17–21]. Combining the above findings with the notion of explorative learning, we suggest that providing participants with extra means of exploration through visual feedback on task-relevant propulsion variables will enhance the motor learning process.

Therefore, the current experiment aims to assess if learners, who actively explore their motor space using visual feedback on propulsion technique variables as guidance, learn more than learners who do not receive any feedback and therefore undergo a natural learning process. We hypothesize that feedback-induced variability will enhance the motor learning process (operationalized as improvement in mechanical efficiency and propulsion technique) in the feedback group more than the natural learning practice. To evaluate the effectiveness of the proposed motor learning training it was chosen to include able-bodied participants who are naïve to wheelchair propulsion. The inclusion of able-bodied participants with similar age and lack of wheelchair experience eliminates potential confounders resulting from trauma or disease, which are often present in the wheelchair-dependent population: e.g. lack of sitting balance or presence of pain. Therefore, the inclusion of able-bodied participants ensures a homogenous group, which will allow to more accurately isolate the effect of feedback-induced variability on the motor learning process.

## Methods

### Participants and Ethics Statement

Thirty-two men participated voluntarily in this study. To compare with earlier research in our laboratory only male subjects were selected. The average age of the participants in the feedback group was 22.9 ± 2.9 years and in the natural learning group 22.8 ± 3.9 years. The average mass of the participants in the feedback group was 82.4 ± 12.5 kg and in the natural learning group 83.4 ± 10.4 kg. The average height of the participants in the feedback group was 1.86 ± 0.05 m and in the natural learning group 1.87 ± 0.08 m. All participants signed an informed consent before the onset of the experiment, following detailed verbal and written information about the character of the study. The protocol of the study was approved by the Local Ethics Committee, of the Center for Human Movement Sciences, University Medical Center Groningen, University of Groningen, The Netherlands. Criteria for inclusion were: being able-bodied and having no previous experience with wheelchair propulsion. The exclusion criterion was: presence of severe medical conditions that could influence parameters measured in the present study, including any musculoskeletal complaints, especially involving the shoulder complex and upper extremities.

### Experimental Setup

**Fig 1:**
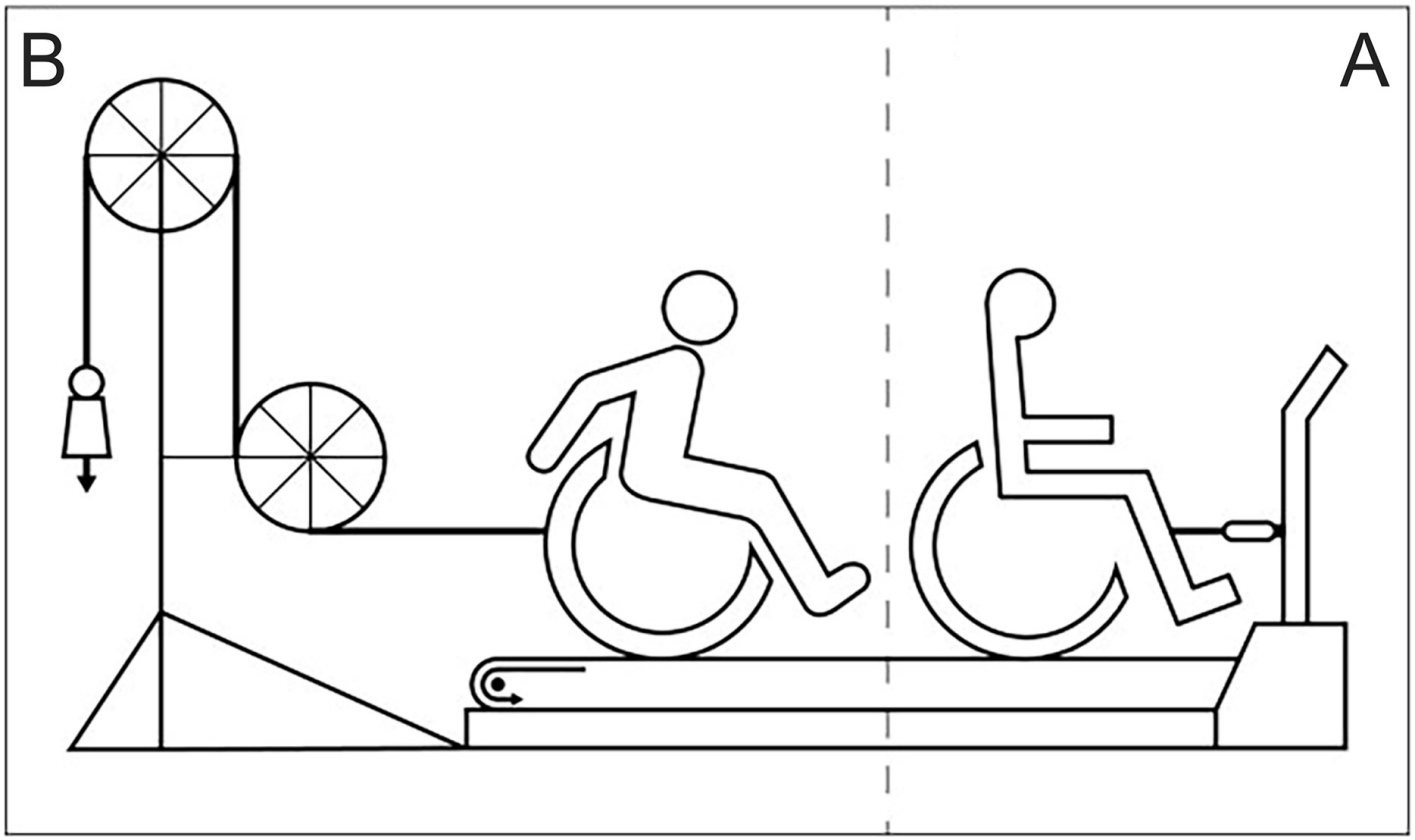
(A) The extra resistance needed to maintain the power output was calculated for each participants individually based on the data acquired from a drag test. (B) Power output was set using the pulley system (figure from Vegter et al. [25]).

**Fig 2:**
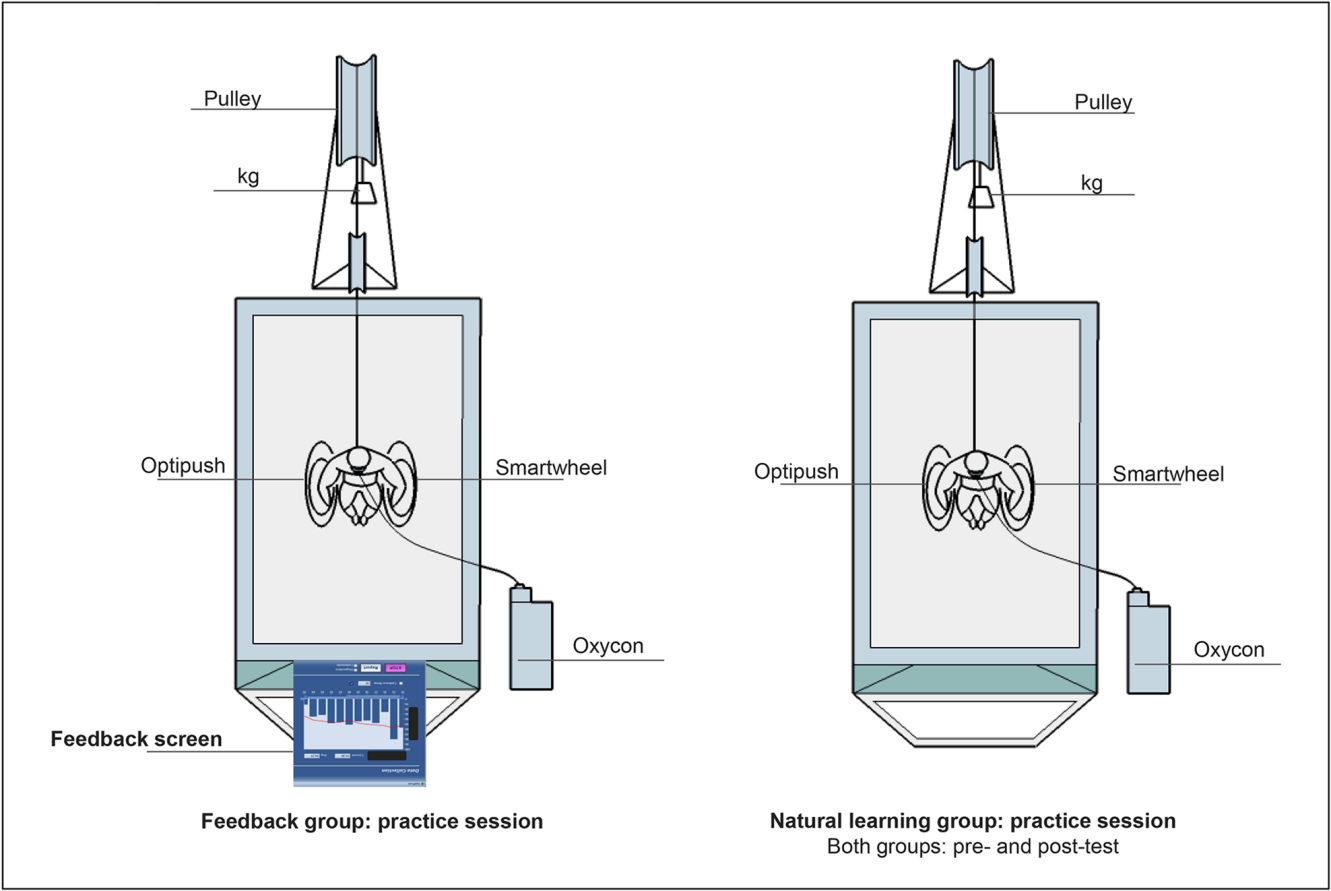
The experimental setup. The setup during practice sessions for the feedback (left side) and the natural learning group (right side). The set up presented on the right side of the figure was also utilized during the pre- and post-test in both groups.

All measurements were performed in an experimental handrim wheelchair (Double Performance BV, Gouda,The Netherlands) placed on a 2.4 m long and 1.2 m wide level motor-driven treadmill (Forcelink b.v, Culemborg The Netherlands). The wheelchair remained unchanged throughout the experiment and for all participants. The aspects concerning the wheelchair-user interface such as seat height, torso height and distance between acromion and axle position were not included in the current study. Tire pressure of the rear wheels was set at 600 kPa during all practice and test sessions. Treadmill velocity was set at 1.11 m/s and power output at 0.24 W/kg body mass throughout the 80 min experiment. The extra resistance needed to maintain the power output was calculated for each participant individually, based on the data acquired from a drag test prior to experimentation (Fig 1A). The drag test, developed by the technical workshop of the Faculty of Human Movement Sciences at the VU University in Amsterdam, measures the rolling resistance, which together with the velocity determines the power output [22, 23]. The extra resistance was added using a pulley system [24] (Fig 1B). The experimental setup is presented in Fig 2.

### Procedure and feedback-induced variability

**Fig 3:**
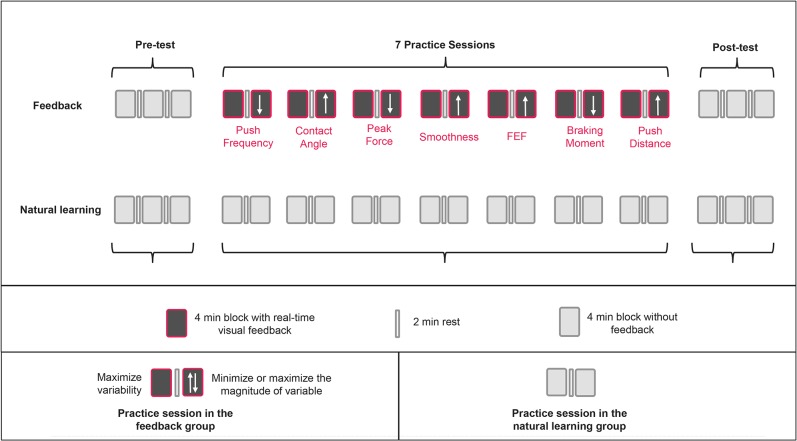
Study protocol for the feedback and the natural learning group. Pre- and posttest consisted of 4 x 3min blocks each. Seven practice sessions consisted of 2 x 4min each. A different propulsion variable at each practice session was presented in the form of real-time visual feedback to the participants in the feedback group. The order of the propulsion variables was counterbalanced over the participants. Participants in the natural learning group practiced without feedback. Last minute of each exercise block was used in the analysis.

17 Participants received visual feedback-based practice (feedback group) and 15 participants received regular practice with no feedback or instruction (natural learning group). Both groups received the same practice dose of 80-min spread over a period of 3 weeks (Fig 3). This protocol duration was chosen since previous research showed that it allows for observing significant changes in mechanical efficiency and propulsion technique [10, 12, 13]. The 80 min dose consisted of a 12 min (3 x 4min, with bouts of 2-min rest between the exercise blocks) pre- and post-test and 7 sessions of 8 min (2 x 4min, with 2-min rest between the exercise blocks) of submaximal handrim wheelchair practice on a motor-driven treadmill. The pre- and post-test were performed without any feedback in both groups (Fig 3).

**Fig 4:**
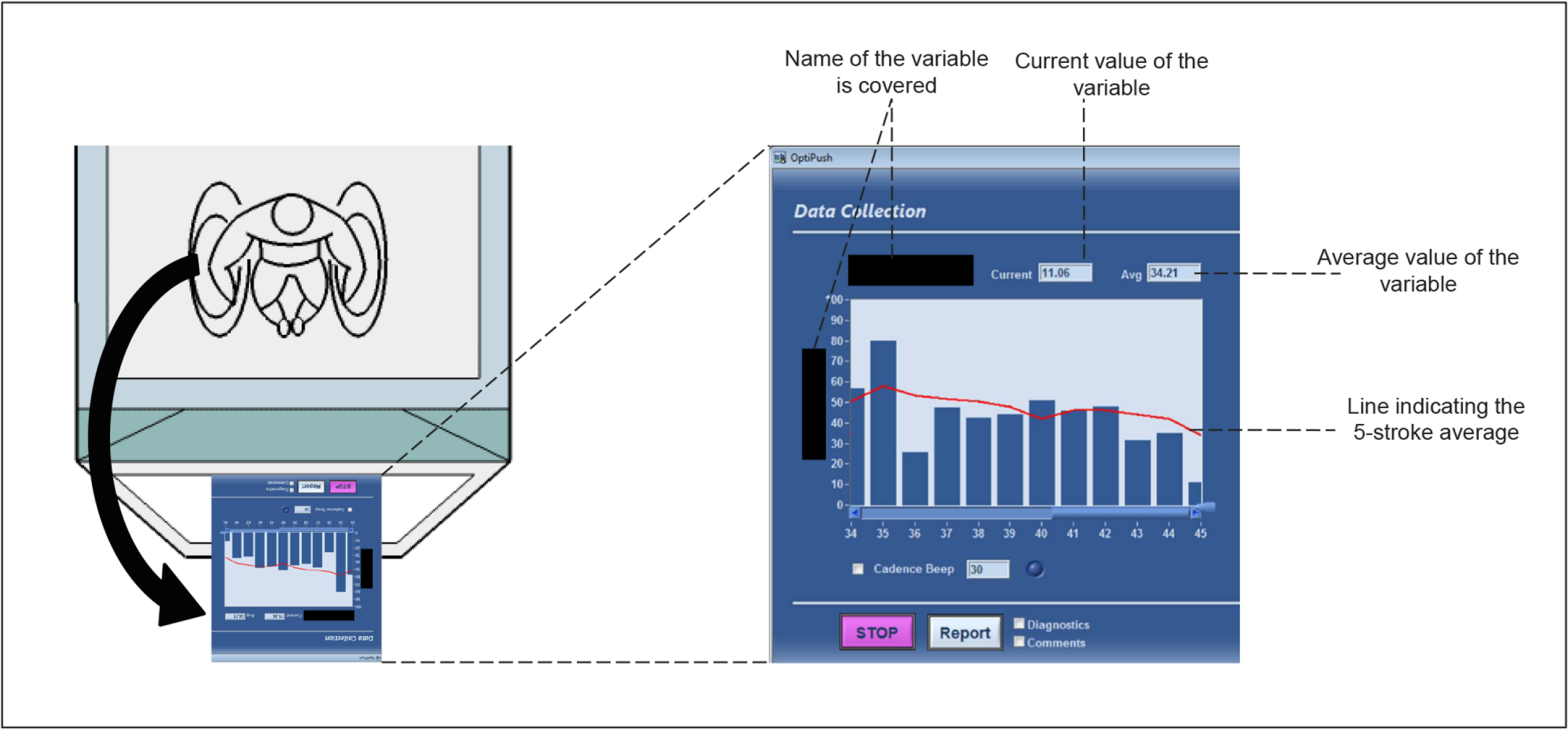
Real-time visual feedback screen. Participants in the feedback group0020received real-time visual feedback on different propulsion variables at each practice session. The black arrow on the left side indicates that forces and torques applied by a person to the handrim were calculated into specific propulsion technique variables and presented real-time on the feedback screen in the form of a bar graph. Participants were informed that they could alter the height of the bars by changing their propulsion technique. The task in the first out of two blocks was to vary the height of the bars on the screen. In the second block the height had to be either minimized or maximized, depending on the propulsion variable.

Counterbalanced over the participants, the feedback group received real-time visual feedback (Fig 4) on seven different propulsion variables (2 x 4min/variable): push frequency, braking moment, contact angle, peak force, push distance, smoothness and fraction effective force (Table 1). The vision ability of the participants in the feedback group was checked by asking the participant to read the average value of the propulsion technique variable (Fig 4), which was presented on the feedback screen, while seating in a wheelchair in the front, middle and at the end section of the treadmill. Visual feedback was provided using software of the instrumented wheel Optipush (MAX Mobility, LLC, Antioch, TN, USA). The visual feedback was presented real-time on a 22” computer screen. The value of the variable is displayed once the start and the end of a cycle is calculated. This provides a slight delay in the feedback. Each propulsion variable was presented to the participants on a 22 inch screen in the form of a bar graph displaying the magnitude of the variable push-by-push. Participants were informed that they could alter the height of the bars by changing their propulsion technique. To increase motor exploration and intra-individual variability, the participants didn’t know which variable they were practicing on during a given practice session. Descriptions or names of any of the seven propulsion variables were not provided before or during the experiment. Participants had to discover their solutions and options themselves. However, participants did receive feedback on the screen about their performance and were encouraged to manipulate the unknown variable to achieve the highest possible variability (1st 4-min block) and to optimize it in the prescribed direction (2nd 4-min block). Before each block, the participants were asked to explain the task in their own words to make sure that they understand the instruction and know how to correctly perform the task. No target line was displayed for the propulsion variables to guide the participants. This way, each participant was given the freedom of exploration without providing additional task constraints.

**Table 1:** The propulsion variables.

Propulsion variable	Unit	Description	Equation	Direction of the manipulation^a^
Push frequency	push/minute	The number of pushes performed during one minute	N_pushes_/Δt	Minimize
Braking moment	Nm	The braking moment applied to the handrim with each push. The sum of braking moment exerted on the handrim during coupling and decoupling of the hand	Σ_end_(i)_:start_(i+1) (Tz · ΔØ)	Minimize
Contact Angle	degrees (°)	The angle measured along the handrim, where subject's hand maintained contact with the handrim during each push	Ø_end_(i)_-_Ø_start_(i)	Maximize
Smoothness	no unit^b^	The ratio of mean to peak force per push	Mean_(start:end)_ (Fx^2^+ Fy^2^+ Fz^2^)^0,5^/Max_(start:end)_ (Fx_2_+ Fy_2_+ Fz_2_)^0,5^	Maximize
FEF	%	The ratio of effective to total force that was applied to the handrim during one push	Mean_(start:end)(_((Tz/r)/((Fx^2^+ Fy^2^+ Fz^2^)^0,5^))·100%	Maximize
Push distance	m	The distance covered with each push	Mean_(start:end)_V·Δt	Maximize
Peak force	N	3d peak force applied to the handrim during one push	Max_(start:end)_ (Fx^2^+ Fy^2^+ Fz^2^)^0,5^	Minimize

^a^ Only applicable for the second block of the practice session in the feedback group.

^b^ Smoothness is calculated by dividing average force (N) by peak force(N).

Abbreviations: t, time(s); start(i), start of the current push (sample); end(i), end of the current push (sample); Tz, torque around wheel axle (Nm); Ø, angle (rad); Fx, Fy and Fz, force components (N); r, wheel radius (m); V, velocity (m/s).

The variables were used in the form of visual feedback to increase the intra-individual variability and as outcome variables to compare the change in propulsion technique between the groups. All variables except cadence were calculated as an average value of all pushes performed during last minute of each practice block. Equations from Vegter et al [12, 25].

### Motor learning

#### Mechanical efficiency

Oxygen uptake (VO2) and respiratory exchange ratio (RER) during steady-state wheelchair propulsion were continuously determined breath-by-breath using Oxycon Pro-Delta (Jaeger, Hoechberg, Germany), which was calibrated before each measurement occasion using Jaeger 5 l syringe, room air and a calibration gas mixture.

Mechanical efficiency was calculated over the last minute of each 4-min block. The equation used to calculate mechanical efficiency was: ME = PO x E ^-1^ x100%, where PO is a power output and E is the energy expenditure, calculated according to:

PO(W)=T(torque(Nm))xAv(Angular velocity(rad/s))

E (W) = (4940 x RER + 16040) x VO_2_ (ml/kg/min) / 60, where RER and VO_2_ are the average values over the last minute of each exercise block [26]. The last minute was chosen to make sure that steady-state propulsion was reached [27]. RER used to calculate the energy expenditure, can only be used as an estimation of the substrate utilization if the participant propels the wheelchair at the steady-state submaximal intensity.

#### Propulsion technique variables

The absolute values of the propulsion technique variables (Table 1) were used to evaluate the effect of practice on the propulsion technique. Applied forces and torques on the hand rim were continuously measured throughout the whole experiment. Software of the instrumented wheel Optipush (MAX Mobility, LLC, Antioch, TN, USA), which measures 3-dimensional forces and torques that a user applies to the handrim, was used to gather data from the right wheel. The data from the left side was collected using a Smartwheel instrumented wheel (Three Rivers Holdings, Mesa, AZ, USA) and could be used to replace missing Optipush data, since the two measurement wheels have high consistency which allows the data to be used interchangeably [25]. The data from the right side was used for the analysis. Both measurement wheels were mounted to the 0.61 m wheels (diameter of the handrim was 0.53 m) with inflatable tires. The measurement frequency of both wheels was set at 200 Hz. The data collected during the last minute of each 4-min block was used for analysis. The output from the measurement wheels was analyzed using custom-written Matlab algorithms [25].

### Statistical analysis

Statistical analysis concerning the data from the practice sessions and the characteristics of the participants was performed using IBM SPSS Statistics version 21.0 (SPSS Inc., Chicago, IL, USA). All data showed normal distribution at baseline, therefore parametric tests were applied. The age and body mass of the participants were compared between the natural learning and the feedback group, using independent t-test, to check for presence of the initial differences.

For the 3 blocks of the pre-test, the 7 practice sessions (2 blocks each) and the 3 blocks of the post-test, the intra-individual variability for each propulsion variable was quantified as the coefficient of variation, calculated over the last minute of each individual block (CV, the ratio of the standard deviation to the mean, CV = σ/μ x 100 (%)) and averaged across the 7 propulsion variables. Finally the CV was averaged across subjects within one group.

In order to determine if the participants indeed increased their intra-individual variability during the practice sessions, a repeated measure ANOVA with session (7 practice sessions) and group (feedback or natural learning) was performed for block 1 and block 2 separately. The group effect was used to determine the difference in variability (CV) between the feedback and natural learning group.

To examine the difference between the two groups over the duration of the experiment, pre- and post-test values of mechanical efficiency, propulsion technique and intra-individual variability were compared using MLwiN version 2.31 (Center for Multilevel Modeling, University of Bristol, Bristol, UK). The data from the 3 pre-test blocks (4 min each, last minute used for the analysis) and from the 3 post-test blocks were compared between the groups. Pre- and post-test were represented in the model as time in minutes. Dummy coding was used to distinguish between the groups (0-feedback; 1-natural learning). Considering the possible influence of the power output on the mechanical efficiency and propulsion technique, it was checked whether there was a difference in the power output between the pre- and the post-test between the groups (time x group effect) and within the groups (time effect). In order to prevent bias, in all cases where relative power output differed between two conditions, it was chosen to correct for it by adding power output to the model.

Significance for the repeated-measures ANOVA was set at p < 0.05 and by use of the Bonferroni correction the significance for the post hoc t-tests watch adjusted for the number of comparisons.

## Results

All participants completed the protocol. There were no differences between the groups at baseline with regard to the demographics.

The relative power output during pre- and post-test was significantly lower (p<0.001) for the feedback group (0.242 ± 0.021 W/kg) compared to the natural learning group (0.248 ± 0.017 W/kg). Power output within the feedback group at pre-test (0.253 ± 0.015 W/kg) was significantly (p<0.001) higher compared to the post-test (0.232 ± 0.021 W/kg). No differences between the pre- and the post-test were seen within the natural learning group.

### Feedback-induced variability

**Fig 5:**
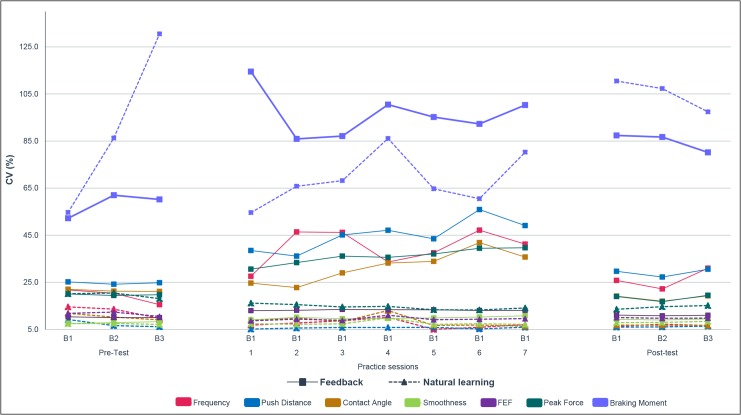
Course of variability (CV) for each propulsion variable. B1, B2 and B3 represent respectively Block 1, Block 2 or Block 3.

**Fig 6:**
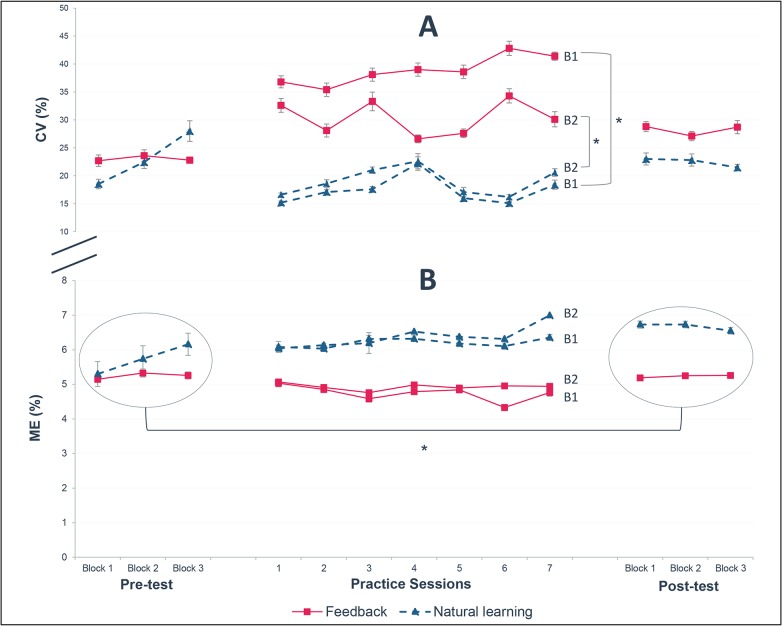
Course of variability (CV) and mechanical efficiency (ME) across the experiment in both groups. (A) Course of variability (mean CV of all seven propulsion variables and standard error) in the feedback and the natural learning group. Participants in the feedback group (n = 17) showed higher variability during both blocks of the practice sessions when compared to the natural learning group (n = 15) (B) Mechanical efficiency (mean and standard error) was lower in the feedback group (n = 17) between pre- and post-test when compared to the natural learning group (n = 15). * indicates a significant difference p<0.05. B1 and B2 represent respectively Block 1 and Block 2 of the practice sessions.

Visual feedback-based practice succeeded in increasing the intra-individual variability during the practice sessions (Fig 5 for individual curves and Fig 6A for the mean of the seven variables). During all the practice sessions (Table 2), the feedback group showed more variability than the natural learning group. This effect was not only visible in the first block where the feedback group received an instruction to perform most variable possible (p<0.001), but also in the second block in which they had to optimize the value of the given propulsion variable (p<0.001).

**Table 2:** Variability (CV) and mechanical efficiency (ME) in the feedback and the natural learning group in the practice sessions.

		Mean ± SD				CV		
		Feedback		Natural learning		Repeated measures ANOVA, group effect		
		CV	ME	CV	ME		P value	F (df, df)
Practice 1								
	Block 1	36.8 ± 22.8	5.03 ± 0.9	15.2 ± 3.0	6.08 ± 1.2	Blocks 1^a^	<0.001	56.304 (1, 26)
	Block 2	32.6 ± 24.5	5.07 ± 1.1	16.6 ± 3.4	6.04 ± 0.7			
Practice 2								
	Block 1	35.4 ± 13.2	4.85 ± 0.9	17.1 ± 5.5	6.03 ± 1.1	Blocks 2^a^	<0.001	36.935 (1, 26)
	Block 2	28.1 ± 12.7	4.91 ± 0.7	18.6 ± 7.6	6.13 ± 0.6			
Practice 3								
	Block 1	38.1 ± 12.8	4.58 ± 0.8	17.6 ± 5.4	6.32 ± 0.7			
	Block 2	33.3 ± 18.3	4.76 ± 0.8	21.0 ± 6.2	6.19 ± 0.7			
Practice 4								
	Block 1	39.0 ± 12.6	4.79 ± 0.6	22.2 ± 12.7	6.32 ± 0.6			
	Block 2	26.5 ± 7.5	4.98 ± 0.7	22.6 ± 13.6	6.53 ± 0.7			
Practice 5								
	Block 1	38.6 ± 13.2	4.84 ± 0.5	16.0 ± 6.7	6.18 ± 0.7			
	Block 2	27.6 ± 8.5	4.89 ± 0.6	17.1 ± 9.0	6.38 ± 0.7			
Practice 6								
	Block 1	42.8 ± 13.9	4.33 ± 0.8	15.1 ± 3.3	6.11 ± 0.5			
	Block 2	34.3 ± 13.9	4.96 ± 0.6	16.1 ± 4.5	6.31 ± 0.3			
Practice 7								
	Block 1	41.4 ± 8.0	4.76 ± 0.9	18.4 ± 9.3	6.36 ± 0.4			
	Block 2	30.1 ± 14.8	4.94 ± 0.7	20.6 ± 7.8	7.00 ± 1.2			

^a^ Comparison of CV between the groups, separately for all blocks 1 and blocks 2 of all practice sessions; CV of all 7 propulsion variables was averaged across each group.

Although the variability in the feedback group showed an increase over the practice sessions, the interaction effect between groups was not significant over time when looking at the pre- and post-test (group x time interaction, p = 0.110) (Table 3)

**Table 3:** Results of multilevel analysis concerning the difference in variability (CV), mechanical efficiency (ME) and propulsion technique variables (Mean ± SD) between the pre- and the post-test between the feedback (n = 17) and the natural learning group (n = 15).

	Feedback			Natural learning			
	Mean ± SD		P value	Mean ± SD		P value	P value
	Pre^a^	Post^a^	Time	Pre^a^	Post^a^	Time	Interaction Time x Group
CV^b^	23.0 ± 10.0	28.2 ± 10.8	0.032	22.8 ± 15.1	22.4 ± 10.6	0.495	0.110
ME	5.25 ± 0.85	5.23 ± 0.59	0.134	5.71 ± 1.34	6.67 ± 0.72	<0.001	0.012
Propulsion variable (unit)							
Frequency (pushes/min)	62.1 ± 18.7	41.5± 13.7	< 0.001	71.3 ± 18.8	52.5 ± 13.8	< 0.001	0.778
Push Distance (m)	1.16 ± 0.28	1.81 ± 0.67	< 0.001	1.04 ± 0.33	1.42 ± 0.38	< 0.001	0.134
Contact Angle (degrees)	66.3 ± 15.4	88.0 ± 16.8	< 0.001	60.0 ± 13.2	77.5 ± 13.4	< 0.001	0.424
Smoothness	0.61 ± 0.04	0.58 ± 0.06	0.138	0.62 ± 0.03	0.60 ± 0.04	0.009	0.823
FEF (%)	69.5 ± 10.1	71.7 ± 9.8	0.694	68.6 ± 10.4	73.6 ± 10.0	0.013	0.246
Peak Force (N)	89.7 ± 30.1	91.9 ± 27.8	0.708	80.1 ± 22.1	76.4 ± 14.2	0.301	0.359
Braking Moment (Nm)	-0.79 ± 0.55	-0.69 ± 0.96	0.134	-0.56 ± 0.81	-0.19 ± 0.20	0.012	0.096

^a^ the average value of 3 blocks

^b^ CV of all 7 propulsion variables was averaged across each group

### Mechanical efficiency

The change in mechanical efficiency across the whole study duration is presented in Fig 6B. As presented in Table 3, the feedback group did not improve the mechanical efficiency over the practice period (p = 0.134). In contrast, the natural learning group improved mechanical efficiency significantly when comparing the pre-and post-test (p<0.001). Moreover, the interaction effect of group x time also reached significance (p = 0.012), indicating that the natural learning group improved the mechanical efficiency in contrast to the feedback group.

### Propulsion technique

No significant differences were found between the groups regarding the change in propulsion technique over time. Both groups significantly decreased the frequency and increased the push distance and contact angle. Although the natural learning group significantly improved smoothness, FEF and braking moment, this effect was not significantly different in the feedback group. The differences in propulsion technique between the pre- and post-test are presented in Table 3.

## Discussion

The aim of the present study was to assess if feedback-induced variability on wheelchair propulsion variables will enhance the motor learning process more than natural learning practice. Motor learning was operationalized as an improvement in mechanical efficiency and propulsion technique variables.

The findings of the present study showed that in the feedback group, intra-individual variability could be successfully increased by means of visual feedback on the propulsion technique variables. However, the increase in feedback-induced variability did not lead to the improvement in the mechanical efficiency. In contrast, the natural learning group did increase the mechanical efficiency. The improvement of mechanical efficiency in the natural learning group that practiced without any external instruction or feedback is in line with other natural learning training studies performed with able-bodied individuals [10–13] and patient populations [28–30].

In contrast to mechanical efficiency, both the natural learning and the feedback group, changed their propulsion technique by for instance decreasing push frequency, increasing push distance and increasing the contact angle, implying a similar improvement in motor learning on these propulsion technique variables. The change in propulsion technique in both groups was in agreement with changes that were previously observed during motor learning studies in wheelchair propulsion [10–13, 31].

Previous research found that changes in propulsion technique due to motor learning, were related to a change in mechanical efficiency [12]. It must, however, be emphasized that in the present study, change in propulsion technique in the feedback group was not linked to the improvement in the mechanical efficiency in contrast to the natural learning group. Even though both groups improved the propulsion technique to a similar extent, only in the natural learning group this change was accompanied by an improvement in mechanical efficiency.

These results suggest that improvement in propulsion technique does not automatically imply an increase in mechanical efficiency. Considering that lower mechanical efficiency in the feedback group was concomitant with the increased variability, it may be that variability was the factor that confounds the relationship between the mechanical efficiency and propulsion technique in the wheelchair propulsion.

The feedback-induced variability practice led to an increase in the intra-individual variability. The pronounced difference in the variability between the groups was visible at all practice sessions and in both blocks. However it presumably interrupted the energy optimization of the motor system. We will discuss several factors that may have contributed to this outcome.

To our knowledge, no other wheelchair propulsion study used real-time visual feedback to target the increase in intra-individual variability. Various studies that targeted an increase in the variability at the task goal changed for instance the target location in a striking task [32] or used various body configurations of the learner [33]. In those experiments, increased variability was actually forced on a learner by the task constraints. In the present study, participants were provided with an opportunity to be variable, which allowed them to independently select the amount of variability that was comfortable for them. Moreover, participants in the present study were instructed to show highest possible variability within the practiced variables that are thought to be task-relevant. Nevertheless, one may argue that variability in current study was not task-relevant as seen from the motor control point of view. Wu et al [16] found that increased task-relevant variability predicts faster learning capability. It may be that participants in current study practiced the total variability, which may have been too unspecific and perhaps did not direct the learner’s exploring capabilities to the most relevant motor solutions. Targeting task-relevant variability by for instance instructing the participants to simultaneously increase variability and optimize the absolute value of the variable may have yielded different results. This possibility needs to be assessed in future studies.

Distinction between total and task-relevant variability shows that variability should not be treated as a single construct. Type of variability should be recognized and considered in the interpretation of the research results. With respect to this, the intrinsic and intervention-induced variability needs to be distinguished [34]. Intrinsic variability is “inherent to the motor system while performing a task” and is naturally exhibited by the participant. Intervention-induced variability on the other hand is introduced in the form of instruction or feedback. It may be that intrinsic variability (variability observed during a natural learning process like in Vegter et al. [13]) and intervention-induced variability (variability introduced by the means of feedback such as in the present study) are distinctly different and, therefore, influence the change in energy efficiency differently. As suggested by Ranganathan and Newell [34], the difference in magnitude between intrinsic and feedback-induced variability might have been responsible for their divergent influence on energy efficiency of the motor system. The intrinsic intra-individual variability measured by Vegter et al. [13] oscillated around 15%, while in our study mean variability in the feedback group was 39% in the first and 30% in the second practice block. This suggests that increasing motor exploration beyond some level may not benefit the motor learning process. It may be that only variability within some range enhances performance. Participants in the present study were naïve to the task of wheelchair propulsion and, therefore, their motor performance may not have been stable yet. It may be that provoking increased variability, especially in the early stages of skill acquisition when performance is not stable, creates dysfunctional movement patterns and is detrimental to learning [34,35].The dysfunctional movement patterns and non-stable motor behavior may have inhibited the optimization of mechanical efficiency in the feedback group.

Another possible explanation for the lack of improvement of mechanical efficiency in the feedback group might be the chosen intervention type. The use of visual feedback can be seen as an extra cognitive task that contributed to higher metabolic energy expenditure during the feedback sessions. However the feedback was not present during the pre- or post-test, which is when the change in mechanical efficiency was evaluated.

Although we did not find a positive influence of variability on the mechanical efficiency, it may be that variability influences other relevant aspects of wheelchair propulsion such as shoulder pain. Rice et al [36] found that the analysis of intra-individual variability allowed making a distinction between pain and no-pain groups, suggesting a link between reduced variability and increased upper-extremity injury risk. The authors recommended investigating whether wheelchair users can be trained to propel the wheelchair in a more variable way in order to decrease the injury risk [36, 37]. The current study showed that an increase in variability in able-bodied participants can be achieved by targeting propulsion variables during feedback-based practice. Possible effect of the feedback-induced variability on the injury risk in wheelchair users is yet to be determined.

The feedback group, next to receiving the visual feedback on their propulsion technique, has also received a brief verbal instruction to show highest possible variability within the practiced variable (1^st^ practice block) and optimize the absolute value of the variable (2^nd^ practice block). The natural learning groups did not receive any verbal instruction. It was purposely chosen not to provide any verbal instruction to the natural learning group. The natural learning protocols in wheelchair propulsion are well researched and show that letting the participants to choose their way of propulsion yields positive effects in propulsion technique and mechanical efficiency [10–13, 31]. In all these protocols, no verbal instruction was provided to the participants. Introducing extra instruction in the natural group in present study would modify the learning process and make the interpretation of the results difficult since observed effect could be an effect of either learning process or the instruction or a combination of both. Therefore, providing verbal instruction to the natural learning group would result in a learning process which could not be described anymore as natural. It has to be acknowledged that the results obtained in the feedback group are the consequence of the added visual feedback on the given variable in combination with a brief standardized verbal instruction.

Finally, a relative homogeneous group of able-bodied participants performed the experiment in a standardized wheelchair without adjustments for the participant’s anthropometry. Therefore, the generalization of the results obtained in this study to patient populations should be done with caution. At the moment we would not advocate to use the tested protocol with patient groups. Future experiments should first further explore the feasibility of increasing the functional component of variability to promote motor learning.

Our study introduced a new experimental approach that, to our knowledge, has not been used before in wheelchair propulsion research: the use of visual feedback to evoke variability. Moreover, present study reveals a possibly complex relationship between propulsion technique and mechanical efficiency that may depend on the intra-individual variability. Yet, it must be noted that the effect of the visual-feedback variability on the mechanical efficiency and propulsion technique is specific to the particular experimental design chosen in this study and should not be generalized to other sorts of feedback-induced variability.

## Conclusion

The feedback group was successful in performing the task with higher intra-individual variability and improved the propulsion technique between pre- and post-test to the same extent as the natural learning group. In contrast, mechanical efficiency remained unchanged in the feedback group but improved between pre- and post-test in the natural learning group.

These results may possibly imply that feedback-induced variability was not beneficial for the motor learning process, but rather hindered the improvement in mechanical efficiency. Moreover since both groups improved propulsion technique but in the feedback group this improvement was not accompanied by the improvement within mechanical efficiency, it can be concluded the mechanical efficiency and propulsion technique are not directly related. It may be that changes in mechanical efficiency and propulsion technique during motor learning process are mediated by other factors such as the amount of the intra-individual variability. This novel finding provides new insights concerning the motor learning process in wheelchair propulsion and it should be considered in the research concerning the relationship between variability and motor learning. Future research should try to replicate the results obtained in the present study on a group of manual wheelchair users (in early rehabilitation), in order to allow to use the results in the development of the clinical interventions.

## Supporting Information

S1 Data Complete Data Set used for data analysis. (XLSX)
